# Toxicity of oxidized phospholipids in cultured macrophages

**DOI:** 10.1186/1476-511X-11-110

**Published:** 2012-09-07

**Authors:** Ute Stemmer, Zsuzsanna A Dunai, Daniel Koller, Gabriel Pürstinger, Elfriede Zenzmaier, Hans P Deigner, Elma Aflaki, Dagmar Kratky, Albin Hermetter

**Affiliations:** 1 Institute of Biochemistry, Graz University of Technology, Petersgasse 12/2, A-8010, Graz, Austria; 2 Department of Mechanical and Process Engineering, Furtwangen University, 78054 Villingen-Schwenningen & Fraunhofer Institute IZI/EXIM, Furtwangen, Germany; 3 Institute of Molecular Biology and Biochemistry, Medical University of Graz, 8010, Graz, Austria; 4 Current address: Department of Pathogenetics, National Institute of Oncology, 1122, Budapest, Hungary; 5 Current address: National Human Genome Research Institute/NIH, Molecular Neurogenetics Section, Bethesda, MD, USA

**Keywords:** Apoptosis, Apoptotic blebs, Macrophages, Acid sphingomyelinase, Ceramide, Atherosclerosis

## Abstract

**Background:**

The interactions of oxidized low-density lipoprotein (LDL) and macrophages are hallmarks in the development of atherosclerosis. The biological activities of the modified particle in these cells are due to the content of lipid oxidation products and apolipoprotein modification by oxidized phospholipids.

**Results:**

It was the aim of this study to determine the role of short-chain oxidized phospholipids as components of modified LDL in cultured macrophages. For this purpose we investigated the effects of the following oxidized phospholipids on cell viability and apoptosis: 1-palmitoyl-2-glutaroyl-*sn*-glycero-3-phosphocholine (PGPC), 1-palmitoyl-2-(5-oxovaleroyl)-*sn*-glycero-3-phosphocholine (POVPC) and oxidized alkylacyl phospholipids including 1-O-hexadecyl-2-glutaroyl-*sn*-glycero-3-phosphocholine (E-PGPC) and 1-O-hexadecyl-2-(5-oxovaleroyl)-*sn*-glycero-3-phosphocholine (E-POVPC). We found that these compounds induced apoptosis in RAW264.7 and bone marrow-derived macrophages. The *sn-*2 carboxyacyl lipid PGPC was more toxic than POVPC which carries a reactive aldehyde function in position *sn-*2 of glycerol. The alkylacyl phospholipids (E-PGPC and E-POVPC) and the respective diacyl analogs show similar activities. Apoptosis induced by POVPC and its alkylether derivative could be causally linked to the fast activation of an acid sphingomyelinase, generating the apoptotic second messenger ceramide. In contrast, PGPC and its ether analog only negligibly affected this enzyme pointing to an entirely different mechanism of lipid toxicity. The higher toxicity of PGPC is underscored by more efficient membrane blebbing from apoptotic cells. In addition, the protein pattern of PGPC-induced microparticles is different from the vesicles generated by POPVC.

**Conclusions:**

In summary, our data reveal that oxidized phospholipids induce apoptosis in cultured macrophages. The mechanism of lipid toxicity, however, largely depends on the structural features of the oxidized *sn-*2 chain.

## Background

Macrophages are prominent cells in atherosclerotic lesions. Within the fraction of lesional macrophages in a proliferating state, a subset becomes apoptotic or necrotic [1]. Studies of advanced atherosclerotic lesions revealed a strong correlation between macrophage cell death and the incidence of plaque rupture associated with acute vascular events [2]. A very early and presumably initiating event in atherogenesis is the focal retention of modified low-density lipoprotein (e.g. oxidized (ox)LDL) in the subendothelial space. Some of the particles that accumulate within macrophages of atherosclerotic lesions are thought to be oxidized either prior to uptake into the arterial wall or subsequently due to intracellular chemical processes, e.g. free radical-mediated modifications. The modified lipoprotein particles can inhibit or induce cell death, depending on the extent of lipoprotein oxidation and lipoprotein dose [3]. In cell culture, apoptosis of macrophages can be initiated by minimally (e.g. by Fe^2+^) modified LDL (mmLDL), in which the lipids but not the apolipoproteins are oxidized [4]. From this observation it can be inferred that the oxidized lipids are largely responsible for the toxic effects of this particle. It has been shown that the toxicity of oxidized phospholipids (oxPL) is increased in cells undergoing ER stress due to other (lipo)toxic agents [5].

mmLDL stimulates acid sphingomyelinase (aSMase) activity within minutes leading to the formation of the second messenger ceramide, which mediates the apoptotic signal in vascular cells including macrophages, endothelial cells and vascular smooth muscle cells [3,6-8]. Ceramide activates several apoptotic signaling pathways. In vascular smooth muscle cells, the stress-induced protein kinases JNK and p38 MAPK have been identified as such components of cells exposed to mmLDL [7].

It has already been shown that ceramide-mediated apoptosis can also be induced in these cells by oxPL [7]. These compounds are generated from polyunsaturated phosphatidylcholines in LDL under the conditions of oxidative stress. Among a plethora of lipid oxidation products, oxPL, such as 1-palmitoyl-2-(5-oxovaleroyl)-*sn*-glycero-3-phosphocholine (POVPC) and 1-palmitoyl-2-glutaroyl-*sn*-glycero-3-phosphocholine (PGPC), are present in mmLDL [9]. They show a high capacity to induce apoptosis in vascular smooth cells [10] and macrophages, especially if the latter cells sense ER stress [5]. Both compounds are oxidation products of phosphatidylcholines containing arachidonic acid in the *sn-*2 position of glycerol. They are characterized by a short fatty acyl chain in position *sn-*2 and a hydrophobic, long-chain, fatty acid in the *sn-*1 position of glycerol. In addition to diacyl-phospholipids, LDL contains the 1-O-alk(en)yl-2-acyl-analogs which are also modified by radical-mediated oxidation [11]. The truncated diacyl and alk(en)ylacyl phospholipids share similar structural features with two highly bioactive lipoprotein and membrane components, namely platelet-activating factor (PAF) and lysolecithin [12,13]. They are more polar than unmodified membrane or lipoprotein phospholipids. Since they contain only one long hydrophobic *sn-*1 acyl or alkyl chain, they exchange easily between tissues, cells, and lipoproteins [14,15].

Exposure of vascular smooth muscle cells to POVPC and to a lesser extent PGPC elicited fast activation of an aSMase in these cells [7,10] pointing to an effect on the protein level. Although both oxPL lead to the same endpoint (apoptosis), their mechanisms of toxicity are likely different. The *sn-*2-aldehydoacyl phospholipid POVPC chemically interacts with the free amino groups of proteins or phospholipids via Schiff base formation [16]. After cellular uptake, most of the lipid is retained in the cell surface. In contrast, PGPC containing a carboxyacyl residue in position *sn-2* can only physically interact with the molecules in its close vicinity and is rapidly internalized by the cells [14,15].

It was the aim of this study to find out whether and to what extent the apoptotic effects of mmLDL are mediated by truncated diacylphospholipids and the corresponding alkylacyl phospholipids in cultured macrophages. For this purpose, we used chemically defined PGPC and POVPC as well as their 1-O-hexadecyl analogs and determined their toxicity in the murine macrophage-like cell line RAW264.7 and bone marrow-derived mouse macrophages (BMM). Although the latter cells proved to be more sensitive towards the oxPL, both cell types showed the same tendencies. The four lipids under investigation mainly induced apoptosis in these cells. PGPC was more toxic than POVPC. The alkylacyl phospholipids and the respective diacyl analogs show very similar activities. Apoptosis induced by POVPC and its alkylether derivative could be causally linked to the activity of aSMase. The more toxic lipids PGPC and its ether analog hardly showed any effect on this enzyme pointing to an entirely different mechanism of lipid toxicity. The higher toxicity of PGPC is underscored by more efficient membrane blebbing from the apoptotic cells producing lots of lipid particles that in turn contain high amounts of oxPL that propagate the toxic phospholipids effects to other cells (and organs).

## Methods

### Materials

Oxidized phospholipids (PGPC and POVPC) were synthesized in our laboratory as previously described [17]. 1-O-alkyl-ether analogs were prepared starting from 1-O-hexadecyl-*sn-*glycero-3-phosphocholine and glutaric acid anhydride as described for the acylation of the 1-acyl-phospholipid [17]. 1-palmitoyl-2-oleoyl-*sn-*glycero-3-phosphocholine (POPC) was synthesized according to the procedure of Hermetter A., Stutz H., Franzmaier R., Paltauf F. (1989) [18]. 1-palmitoyl-*sn-*glycero-3-phosphocholine (PLPC) was purchased from Bachem (Bubendorf, Switzerland). Chemicals for gel electrophoresis were from BioRad Laboratories (Hercules, CA), unless otherwise noted. NB19 was kindly provided by Dr. Hans-Peter Deigner (Frauenhofer EXIM/CEOS, Rostock, Germany). Organic solvents and all other chemicals were purchased from Carl Roth (Karlsruhe, Germany), Sigma-Aldrich (Steinheim, Germany) or Merck (Darmstadt, Germany). Tissue culture materials were obtained from Sarstedt (Nürnbrecht, Germany) or Greiner (Kremsmünster, Austria). Dulbecco’s modified Eagle medium (DMEM, 4,5 g/l Glucose) with and without phenol red and heat-inactivated fetal bovine serum, Vybrant® MTT Cell proliferation Assay kit (V-13154), Vybrant® apoptosis assay kit#2 (V-13241), F4/80 antibody and matching isotype control and staurosporine were from Invitrogen (Leek, Netherlands). PBS and cell culture supplements were obtained from PAA (Linz, Austria) unless otherwise indicated. “Fluids” for flow cytometry, FACS tubes, solutions and cells strainers were from BD Biosciences (Heidelberg, Germany).

### Cell culture

The macrophage-like cell line RAW264.7 (ATCC No. TIB-71, American Type Culture collection, Rockville, MD, USA) was routinely grown in DMEM (4,5 g/l glucose, 25 mM HEPES, 4 mM L-glutamine, without sodium pyruvate) supplemented with 10% heat-inactivated fetal calf serum (FCS) and 100 U/ml penicillin/streptomycin at 37°C in humidified CO_2_ (5%) atmosphere. Bone marrow-derived macrophages (BMM) were isolated according to a slightly modified standard protocol provided by Invitrogen (Leek, Netherlands). Briefly, femur and tibia of C57Bl/6 mice were separated and placed on ice in sterile PBS. The excess muscle was removed and the ends of the bones were cut off on both sides. Cells were washed out with a 26-G needle attached to a sterile syringe, filled with 3 ml DMEM (4,5 g/l D-glucose, 4 mM glutamine, 110 mg/l sodium pyruvate) and transferred via a cell strainer into a 50 ml Falcon tube. Subsequently the cell suspension was centrifuged at 500×g for 15 minutes and the supernatant was discarded. The cell pellet was carefully resuspended in DMEM supplemented with 10% LPDS and 500 U/ml penicillin/streptomycin and transferred into a culture flask (175 cm^2^/3 mice) followed by incubation at 37 °C in a humidified 5% CO_2_ atmosphere for 24 h. Nonadherent cells were removed the next day, counted and resuspended in DMEM containing 10 ng/ml MCSF (R&D Systems, Minneapolis, USA). Cells were seeded in 96 well plates (10^6^ cells/100 μl) for the MTT assay or in 24 well plates (4*10^6^ cells/500 μl) for apoptosis assays. After 3 and 5 days the medium was replaced with fresh, supplemented DMEM containing 10 ng/ml MCSF.

### Assessment of BMM differentiation

Monocyte differentiation was proven with rat anti-mouse F4/80-R-PE antibody and rat anti-mouse IgG2a R-PE isotype control using flow cytometry. The former monoclonal antibody reacts with the mouse F4/80 antigen, which is a macrophage-specific glycoprotein. Monocyte differentiation was analyzed on the first and seventh day using RAW264.7 macrophages as a reference. 10^6^ cells were transferred into a FACS tube and centrifuged at 300×g for 3 minutes. Cells were resuspended in PBS containing 2 mg/ml glucose. 2,5 μg/ml of the antibody or the isotype control were added followed by incubation at room temperature in the dark for 15 minutes. Stained samples were analyzed using a FACSCalibur instrument (BD Biosciences, Heidelberg, Germany). The red fluorescence emission was measured on channel FL2 above 575 nm upon excitation with a 488 nm laser. Analysis was performed on the amplitude (heigh, H) of the fluorescence signal in log scale. The percentage of differentiated cells was calculated using WinMDI 2.8 software. A content of 90-95% differentiated (F4/80 positive) cells was considered as appropriate for further experiments.

### Incubation of cells with oxPL

Aqueous lipid dispersions containing 0–200 μM oxPL, POPC or PLPC were prepared using the ethanol injection method [19]. Cells were incubated with lipid dispersions in PBS or culture media without phenol red containing 0-10% (v/v) serum concentrations. The final ethanol concentration in the incubation mixtures did not exceed 1% (v/v) of total volume. Culture media or PBS containing the same ethanol concentration was routinely used as controls.

### MTT viability assay

To determine the cytotoxic effect of oxPL in macrophages Vybrant® MTT Cell proliferation Assay kit was used according to the manufacturer’s recommendations. The MTT assay involves the conversion of the water soluble MTT (3-(4,5-dimethylthiazol-2-yl)-2,5-diphenyltetrazolium bromide) to an insoluble formazan by viable cells. The formazan is solubilized, and its concentration is determined from the optical density at 570–600 nm. The protocol was optimized for RAW264.7 and BMM according to cell number, MTT concentration and incubation times. In brief, 1,25*10^5^ RAW264.7 cells or 10^6^ monocytes were seeded in 96 well plates in culture medium with varying FCS or LPDS concentrations. Monocytes were differentiated before incubation as described above. RAW264.7 cells were left in the wells for 2–3 h to ensure attachment to the substrate. The medium was removed and the lipid dispersions or control substances (1% v/v EtOH, 2,5 mM H_2_O_2_, 1 μM staurosporin) in PBS or culture medium were added. After incubation, the lipid-containing medium was replaced by 100 μl fresh medium and 10 μl MTT (1 mg/ml stock) solution were added prior to incubation at 37°C in a humidified 5% CO_2_ atmosphere for 2 h. Subsequently, 100 μl 10% (w/v) SDS in 0,01% (v/v) HCl was added. The cells were incubated under the same conditions for 4 h. The mixture was solubilized to homogeneity and optical density was measured at 595 nm using an Anthos plate reader driven by WinRead 2.3 software.

### Flow cytometric apoptosis assay

Samples were prepared as described for the MTT assay (see above), except for the cell number and plate format. Specifically, 6,5 *10^5^ RAW264.7 or 4*10^6^ monocytes were initially seeded in a 24 well plate (volume 3 ml). After incubation, cells were harvested by scraping and washed with cold PBS containing 2 mg/ml glucose prior to resuspension in Annexin V binding buffer. 3*10^5^ cells/100 μl were transferred into a FACS tube. Five μl AlexaFluor®488 Annexin V and 5,5 μl propidium iodide (PI; 1 mg/ml stock solution) were added and allowed to incubate at room temperature in the dark for 15 minutes. Prior to FACS measurement, samples were diluted in 400 μl PBS containing 2 mg/ml glucose, gently mixed and kept on ice until analysis. Stained samples were then analyzed using a FACSCalibur flow cytometer (BD Biosciences, Heidelberg, Germany). The green and red fluorescences were measured at 530 nm and 575 nm, respectively (excitation: 488 nm laser). The [FSC,FL3H] diagram was applied for gating out the debris and the gated cells were analyzed in [FL1H, FL2H] log scale dot plots. Auto-fluorescence of the cells positioned in first decade, and compensation for AlexaFluor®488 fluorescence in FL2 channel was applied. The percentage of apoptotic cells (AnnexinV-positive), necrotic cells (PI-positive) and late apoptotic/early necrotic cell (double-stained) were calculated using WinMDI 2.8 software package. Apoptotic effects of 1-O-alkylether lipids and NB19 were compared with PGPC and POVPC-induced apoptosis under the same conditions except for the cell number. For these experiments, cells were left overnight in the multi-well plate and as a consequence, cell numbers were twice as high. Cells were pre-incubated with 10 μM NB19 in order to determine the effect of aSMase on apoptosis. NB19 was dissolved in EtOH before addition to the culture medium (10 nmol NB19/ml) for 30 minutes. The final ethanol concentrations in the incubation mixtures did not exceed 1% (v/v) of total volume.

### Morphological studies

Monolayer cultures of RAW 264.7(1x10^5^) cells were seeded in chamber slides (Imaging chamber CG 8 wells, PAA, Linz, Austria) and grown to 60–80% confluency over night. Cells were then incubated with 300 μl aqueous dispersions of 50 μM oxPLs or reference compounds (1% v/v EtOH) in DMEM without phenol red under low serum conditions (0,1% FCS) for 2 h. After incubation cells were observed with an Axiovert 35 inverted microscope (magnification: 1000 x) equipped with a CCD camera, driven by AxioVision software package (Carl Zeiss, Germany).

### Proteins of apoptotic blebs

RAW264.7 macrophages were grown to 80% confluency in Petri-dishes (10 cm diameter) and incubated with an aqueous dispersion of 50 μM oxPL or 1% (v/v) EtOH in DMEM under low serum conditions (0,1% FCS) for 18 h. Membrane vesicles and apoptotic blebs were harvested as described previously [20]. Briefly, culture supernatants containing the membrane vesicles were isolated and cleared from debris and detached cells by centrifugation at 500×g for 10 minutes. Blebs were isolated by ultracentrifugation (100000×g for 90 minutes at 4°C). The pellet was washed and resuspended in 30 μl PBS prior to protein determination using a plate assay according to the method of Bradford [21]. Twenty μl of pellet suspensions or supernatants (20 μg protein) were separated by SDS gel electrophoresis (4,5% stacking gel, 10% resolving gel) as previously described [22]. Total proteins were stained with Sypro Ruby™ and detected using a BioRad laser scanner (Ex. 488 nm, Em. 530/30 nm).

### Time-dependent stability of oxPL

Stability of oxPL in serum-containing media was determined as previously described [10]. Solvent was removed from 100 μM oxPL under a stream of nitrogen. Two hundred μl DMEM containing 10% or 0,1% FCS were added followed by incubation under shaking (575 rpm) for 2, 4, 6 or 20 h. After incubation, phospholipids were extracted with chloroform/methanol 2/1 (v/v) at room temperature. The organic phase was removed under a gentle stream of nitrogen. The lipids were then dissolved in chloroform/methanol 2/1 (v/v) and analyzed by thin-layer chromatography on silica plates. The mobile phase was chloroform/methanol/acetone/glacial acetic acid/water 20/40/10/10/10 (v/v/v/v/v) for PGPC and E-PGPC and chloroform/methanol/water 50/30/10 (v/v/v) for POVPC and E-POVPC, respectively. Lipid spots were detected with molybdenum blue reagent, which specifically stains phospholipids [23].

### Acid sphingomyelinase activity

Cultured RAW264.7 macrophages were harvested and counted using Countess System (Invitrogen). 1,3*10^6^ cells were plated in 6 well plates followed by incubation at 37°C in a humidified 5% CO_2_ atmosphere for 24 h. Subsequently, cells were washed with DMEM containing 0,1% FCS. OxPL containing media was prepared by adding ethanolic lipid solutions to DMEM at room temperature under stirring. Cells were incubated with 25 μM oxPL in DMEM at 37°C in a humidified 5% CO_2_ atmosphere for 0–15 minutes. Control cells were incubated with 1% ethanol (v/v) in DMEM under the same conditions. After incubation, all steps were carried out at 4°C. Cells were scraped into 1 ml PBS and the cell suspensions were transferred into 15 ml Falcon tubes followed by centrifugation at 1500 rpm for 10 minutes. The supernatant was discarded and the cell pellet was resuspended in 50 μl acid lysis buffer for the determination of aSMase activity as described previously [24]. Briefly, protein concentration of the cell lysates was determined using a plate assay according to the method of Bradford [21] and aliquots containing 20 μg protein were incubated with NBD-sphingomyelin substrate as described [24]. Substrate and product were separated by TLC. The fluorescent lipids were quantified with a CCD camera (Herolab, Vienna) (excitation wavelength: 365 nm) using EasyWin software.

### Statistical analysis

Results are expressed as means +/− standard deviation (SD). Two-tailed unpaired Student’s *t*-test was used to determine the significance of the differences. p-Values ≤ 0,05 were considered significant.

## Results

**Figure 1:**
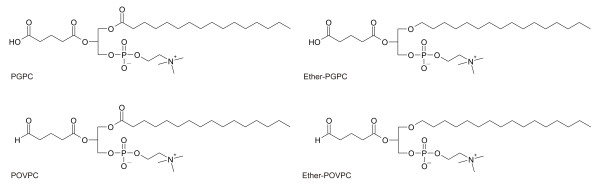
**Chemical structures of oxidized phospholipids.** PGPC: 1-palmitoyl-2-glutaroyl-*sn-*glycero-3-phosphocholine. Ether-PGPC: 1-O-hexadecyl-2-glutaroyl-*sn-*glycero-3-phosphocholine. POVPC: 1-palmitoyl-2-(5-oxovaleroyl)-*sn-*glycero-3-phosphocholine. Ether-POVPC: 1-O-hexadecyl-2-(5-oxovaleroyl)-*sn-*glycero-3-phosphocholine.

The oxPL PGPC and POVPC are components of oxLDL which is causally involved in the onset and progression of atherosclerosis [25]. We have already shown that the toxicity of oxLDL in cultured vascular smooth muscle cells is largely due to its oxPL components. PGPC, POVPC and oxLDL induce apoptosis in these cells, which is mediated by aSMase generating the second messenger ceramide [7]. It was the aim of this study to find out whether and to what extent PGPC and POVPC (Figure 1) induce (programmed) cell death in macrophages, which is a hallmark in atherogenesis.

**Figure 2:**
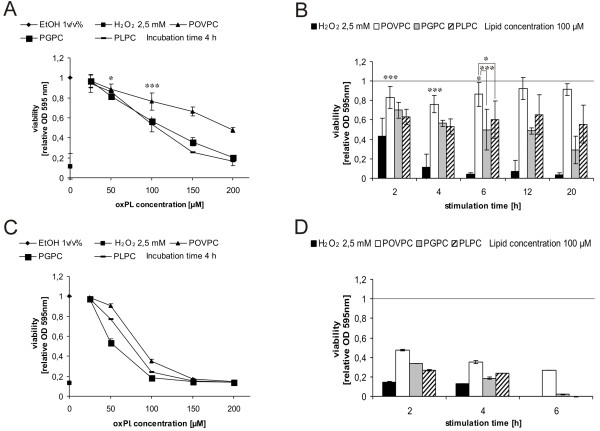
**Effects of oxPL on viability of RAW264.7 cells and BMM.** RAW264.7 cells (Panel **A** and **B**) and BMM (Panel **C** and **D**) were incubated with lipid dispersions in DMEM under low serum conditions (0,1% FCS) using various lipid concentration and incubation times. Cell viabilities were determined using the MTT assay described under materials and methods. Indicated values are relative viabilities (viabilities of control cells were set 1). Results obtained with RAW264.7 cells are means of 6 replicates out of three independent experiments. Results obtained with BMM (isolated and pooled from femurs and tibias of 3 or 5 mice, see materials and methods) are means of 4 replicates out of two independent experiments. Significance was determined by Student’s *t*-test (two tailed, unpaired). * p ≤ 0,05, *** p ≤ 0,005.

For this purpose, we used the established RAW264.7 macrophage-like cell line and cultured murine BMM. In a pre-screen, the effects of both oxPL on cell viability were studied using the photometric MTT assay (Figure 2). From the decrease in optical density of the marker dye it can be concluded that both compounds decreased the viability of RAW264.7 cells in a concentration- and for PGPC in a time-dependent manner (Figure 2A and B) with PGPC being more toxic than POVPC. Notably, BMM were more sensitive towards the oxPL than RAW264.7 cells (Figure 2C and D).

**Figure 3:**
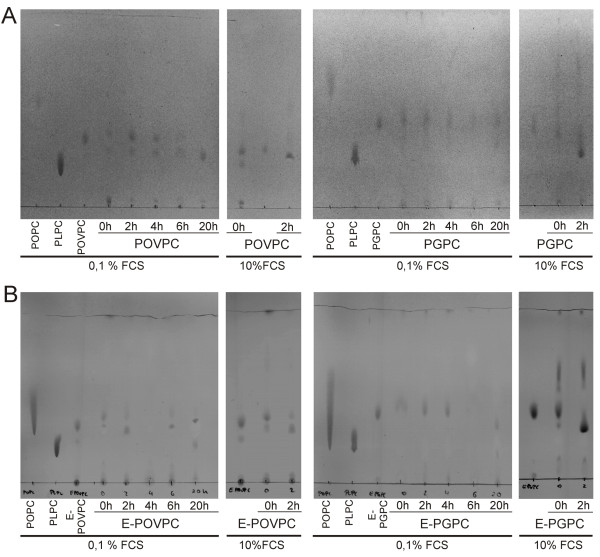
**Time-dependent stability of oxPL in culture media under different serum conditions.** OxPL were incubated in DMEM containing 0,1% or 10% serum for different time periods. Subsequently, lipids were isolated by solvent extraction and separated by TLC. Mobile phase for PGPC (Rf = 0,43) and E-PGPC (Rf = 0,36), POPC (Rf >0,30) and PLPC (Rf = 0,18) was chloroform/methanol/acetone/glacial acetic acid/water 20/40/10/10/10 (v/v/v/v/v). For POVPC (Rf = 0,35) and E-POVPC (Rf = 0,32), POPC (Rf >0,25) and PLPC (Rf = 0,15) the mobile phase was chloroform/methanol/water 50/30/10 (v/v/v) Under high serum conditions (10% FCS), POVPC and PGPC were converted to lysophospholipids. Under low serum conditions (0,1% FCS), PGPC was stable for at least 6 h, whereas POVPC started getting degraded immediately (Panel **A**). Notably, the major amount of oxPL stayed intact during the incubation times used for cell experiments. The 1-O-alkyl ether analogs showed the same stabilities as their acyl counterparts (Panel **B**).

Lysophosphatidylcholine (PLPC) showed comparable effects on viability as PGPC (Figure 2). PLPC and the oxPL show similar structural features since they contain only one long-chain fatty acyl residue in position *sn-*1 and a polar group in position *sn-*2 of glycerol. Since PLPC is formed upon hydrolytic degradation of PGPC and POVPC (see also Figure 3), it can be expected that it contributes to the toxicities of these lipids to some extent, especially after long incubation times.

**Figure 4:**
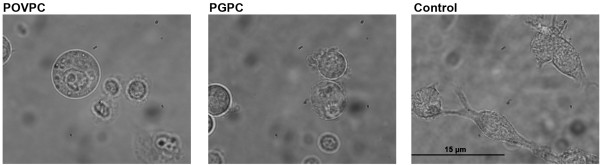
**Morphological changes in RAW264.7 macrophages.** Photomicrographs (magnification: 1000x) of RAW264.7 cells after incubation with POVPC and PGPC in DMEM containing 0,1% FCS for 2 h. Control cells were incubated with 1% v/v EtOH. Signs of cellular damage are cell detachment, morphological changes and membrane vesicle formation.

Cell damage detected by the MTT assay is in line with the morphological changes that are observed under the microscope (Figure 4). Upon treatment with oxPL RAW264.7 cells show morphological changes such as rounding and shrinking and are eventually released from the solid substratum.

**Figure 5:**
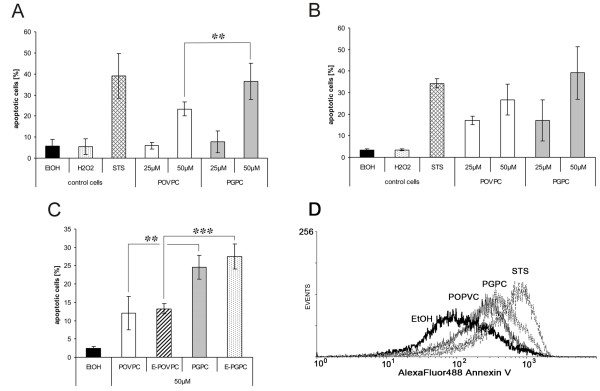
**Apoptotic effects of oxPL in RAW264.7 cells and BMM.** Cells were incubated with the indicated concentrations of oxPLs in media under low serum conditions (0,1% FCS) for 4 h and analyzed by flow cytometry (see materials and methods). The fraction of apoptotic cells was determined from Annexin V staining of externalized phosphatidylserine. Control cells were incubated with 1% v/v Ethanol (EtOH). 2,5 mM H_2_O_2_ or 1 μM staurosporin (STS) in the incubation media, which were used as reference agents inducing necrosis or apoptosis, respectively. Panel **A**: 50 μM POVPC and PGPC induce apoptosis in RAW264.7 macrophages. PGPC is a more potent inducer of cell death than POVPC under these conditions. Results are means of 6 replicates out of three independent experiments. Panel **B**: 50 μM PGPC and POVPC induce apoptosis in BMM, which are slightly more sensitive to the oxPL compared with RAW264.7 macrophages. Results are means from one representative experiment of 4 replicates of one pooled sample (BMM isolated and pooled from femurs and tibias of 5 mice, see materials and methods). Panel **C**: 50 μM 1-O-alkyl ether analogs of PGPC and POVPC (E-PGPC and E-POVPC, respectively) induce apoptosis in RAW264.7 macrophages. Panel **D**: Representative histogram of RAW264.7 cells after exposure to 1% v/v Ethanol (EtOH), 1 μM staurosporin (STS), POVPC or PGPC. Experimental conditions as described under Panel A. All Results are expressed as means +/− SD. Significance was determined by Student’s *t*-test (two tailed, unpaired). ** p ≤ 0,01, *** p ≤ 0,005.

The detrimental effects of both oxPL on cell viability are associated with an increase in apoptosis. Programmed cell death under the influence of PGPC and POVPC was determined from cell staining with fluorescent Annexin V followed by FACS analysis. In addition, the capacity of the oxPL to induce necrosis was measured in the same experiment using PI as dye that stains the DNA in cells with permeabilized membranes. Staurosporine and hydrogen peroxide were used as control toxins to assess apoptosis and necrosis, respectively. PGPC and POVPC induced apoptosis in both cell types with PGPC being again more toxic than POVPC (Figure 5A and B). Cells incubated with either oxPL hardly showed any signs of necrosis (data not shown). As observed before, BMM were more sensitive to the oxPL than RAW264.7 macrophages. We also studied apoptosis of both cell types under the influence of the 1-O-alkyl-2-acyl analogs of PGPC and POVPC (Figure 1). The respective compounds are oxidation products of ether choline phospholipids that are also found in LDL and cell membranes of animals and humans [26]. The ether analogs of PGPC and POVPC and their diacyl counterparts showed very similar apoptotic effects in RAW264.7 cells, with the PGPC analog being more toxic than the POVPC analog (Figure 5C). It is obvious that the toxicities of the diacyl and alkylacyl oxPL depend on the small structural difference in the oxidized *sn*-2 acyl chain. The (ether) POVPC contains an aldehyde group which can react with the amino groups of phospholipids and proteins, whereas PGPC can only undergo physical interactions with the molecules in its close vicinity. As a consequence, cellular lipid uptake, membrane effects, signaling platforms and the outcome, cell death, are different.

Toxicity of oxPL in macrophages depends on lipid stability inside the cells and in the culture medium prior to cellular uptake. The cell viability and apoptosis experiments were performed under low (0,1%) serum conditions. In order to determine the stability of the oxPL, PGPC, POVPC and their ether analogs were incubated in the culture medium, followed by solvent extraction and TLC analysis. Under low serum conditions (0,1% FCS), POVPC (Figure 3A) and ether-POVPC (Figure 3B) were stable for 2 h, whereas PGPC (Figure 3A) and ether-PGPC (Figure 3B) remained intact for 6 h. This difference can help explain at least in part the higher toxicities of the PGPC versus the POVPC-derived lipids. Addition of 10% serum to the culture medium efficiently catalyzed the hydrolysis of PGPC, POVPC (Figure 3A) and their ether analogs (Figure 3B) to the formation of PLPC. After 2 h of incubation, the entire amount of lipid has gone under these high serum conditions.

**Figure 6:**
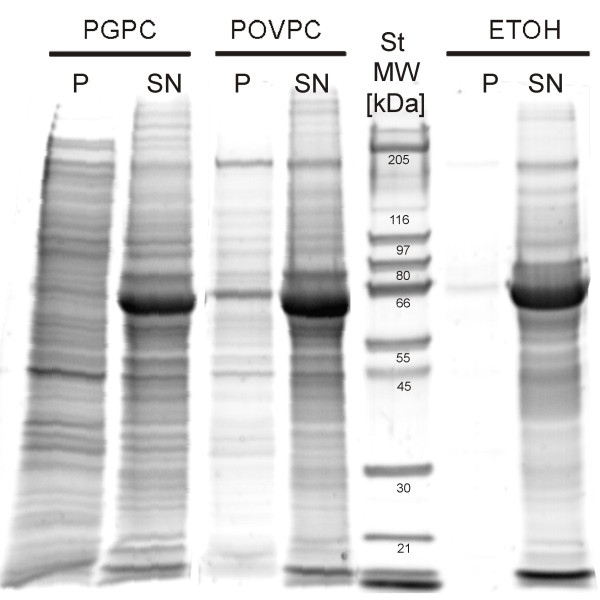
**Protein pattern of apoptotic blebs.** RAW264.7 cells were incubated with 50 μM PGPC or POVPC or 1% (v/v) EtOH under low serum conditions (0,1% FCS) for 18 h. Membrane vesicles were isolated by ultracentrifugation and resuspended in PBS. Proteins of pellets (P) and supernatants (SN) obtained after ultracentrifugation were separated by SDS-PAGE as described in materials in methods section. Vesicles produced by POVPC and PGPC show slightly different protein patterns. More vesicles protein is released under the influence of PGPC.

Apoptosis is associated with a series of profound changes in cellular structure and integrity. Membrane blebbing, the release of vesicular bodies (blebs) from the plasma membrane, is one of the hallmarks of this process [27-29]. To measure the capacity of the oxPL to induce membrane blebbing in RAW264.7 macrophages, cells were incubated with 50 μM PGPC or POVPC for 18 h (Figure 6). The vesicle pellets (P) were isolated from the supernatant (SN) by ultracentrifugation and analyzed for their protein patterns and contents. The protein patterns of the apoptotic vesicles (P) slightly depended on the oxPL. However, the efficiency as determined from the protein amount in the membrane fraction (P) differed to a great extent depending on the lipid. Concerning the protein content we found that PGPC led to a much more pronounced release of apoptotic vesicles than POVPC.

**Figure 7:**
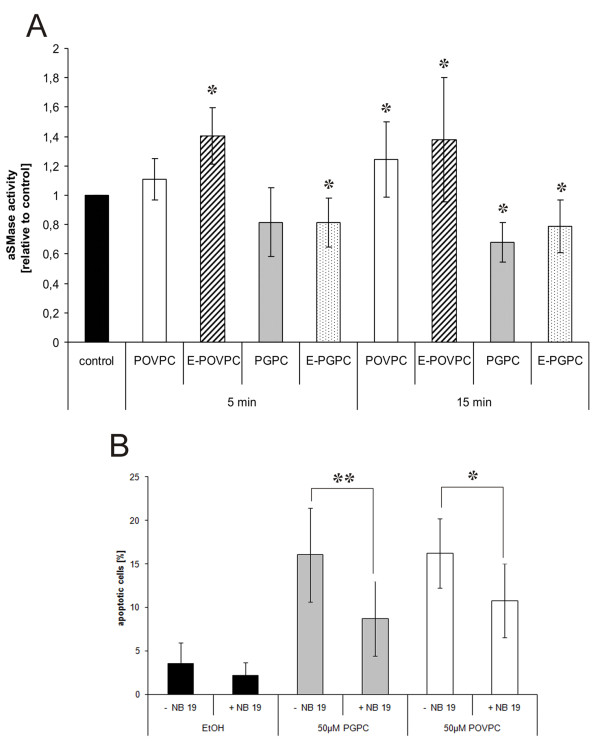
**Effects of oxPL on aSMase activity and aSMase-mediated apoptosis.** Panel **A**: RAW264.7 cells were incubated with DMEM containing 25 μM of lipid or 1% v/v EtOH (control) for 5 or 15 minutes. Cells were harvested and lysed and sphingomyelinase activity was determined as described under materials and methods. Results are shown as relative values (folds of controls). The absolute enzyme activities of the control cells in the individual experimental series ranged from 4 to 8 pmol/min/μg protein. POVPC and E-POVPC increase aSMase activity within 5 minutes. PGPC and E-PGPE had a slightly inhibitory effect on enzyme activity as compared to the control. Panel **B**: RAW264.7 cells were incubated with 50 μM oxPLs in media under low serum conditions (0,1% FCS) for 4 h and analyzed by flow cytometry. The fraction of apoptotic cells was determined from Annexin V staining of externalized phosphatidylserine. Control cells were incubated with 1% v/v EtOH with or without NB19 in the incubation media. Inhibition of aSMase with NB19 (10 nmol/ml) leads to significant decrease in apoptosis induced by POVPC and PGPC. NB19 alone had no significant effect on cell viability. Results are means of 4 replicates out of 3 experiments. Data are expressed as means +/− SD relative to the control. Significance was determined by Student’s *t*-test (two tailed, unpaired). * p ≤ 0,05, **p ≤ 0,01.

In a previous study, we found that POVPC and (to a lesser extent) PGPC-induced apoptosis was mediated by the fast activation of an aSMase in cultured vascular smooth muscle cells [7]. This enzyme generates ceramide from sphingomyelin, which propagates the apoptotic signal. The data presented in this work provide evidence that both oxPL induce apoptosis also in cultured macrophages, but the role of aSMase seems to be different in the latter cells. POVPC and ether-POVPC activate aSMase within minutes (Figure 7A). The ether-POVPC stimulates the enzyme more efficiently. This result is in line with the slightly higher, toxicity of the ether-POVPC. FACS analysis of apoptosis in RAW264.7 macrophages provided evidence that aSMase activity is directly linked to the toxicity of POVPC. Pre-incubation of the cells with an enzyme inhibitor (NB19) reduced the amount of apoptotic cells after exposure to the oxPL (Figure 7B). NB 19 did not influence the basal level of apoptosis in control cells without (ether-) POVPC. PGPC and ether-PGPC show an opposite effect as they inhibit aSMase in macrophages (Figure 7A). We have evidence that PGPC utilizes enzymes of the *de novo* pathway for ceramide production and signaling (unpublished). Interestingly, inhibition of aSMase expression by NB19 protects cells against the toxicity of PGPC (Figure 7B), although there is no direct effect of the oxPL on enzyme activity. According to these data, there must be at least one indirect relationship between aSMase and PGPC-induced cell death. This open question will be subject to further clarification.

## Discussion

Apoptosis of macrophages in the arterial wall is a hallmark in the development of atherosclerosis [29]. Accumulation of apoptotic cells along with a transition to cell necrosis contribute to the destabilization of atherosclerotic plaques followed by plaque rupture and thrombus formation [30]. The endpoint of the development of this chronic disease may be myocardial infarction or stroke. From *in vitro* experiments it is known that the interaction of oxidized lipoproteins with cultured macrophages is responsible for the various facets of cell fate under pathophysiological conditions [25]. oxLDL, characterized by a high content of modified lipid and protein components, is incorporated into the cells via scavenger receptors without regulatory feedback mechanisms. As a consequence, lipids accumulate inside the cells and foam cells are formed. In mmLDL, a significant fraction of cholesterol and (phospho) lipid esters containing polyunsaturated fatty acids are modified or fragmented, whereas apolipoprotein B (apoB) is hardly affected [29]. These particles are also very toxic to macrophages and other cells of the vascular wall, depending on the extent of lipoprotein modification, dose and incubation time. mmLDL is still recognized by the apoB receptor, but its toxicity does not depend on receptor-mediated uptake of the entire particle [31]. OxPL and especially those containing fragmented acyl chains are more polar and can be efficiently transferred to cell plasma membranes through the aqueous phase [14,15]. Sustained exposure to mmLDL induces apoptosis in cultured vascular cells and macrophages [3,4,7], which is mediated by the activation of an aSMase generating the apoptotic lipid messenger ceramide. We have already shown that the truncated phospholipids PGPC and POVPC mimic the toxic effects of mmLDL in cultured vascular smooth muscle cells [7]. Here we provide evidence that both oxPL induce apoptosis in cultured macrophages. In these studies, we investigated RAW264.7 macrophage-like cells and BMM. The tendencies of PGPC and POVPC to induce apoptosis were the same in both cell types with BMM being slightly more susceptible to oxPL toxicity than RAW264.7 macrophages. Despite the fact that primary bone marrow-derived cells were included in our study it has to be noted that the results obtained *in vitro* cannot be directly translated in vivo, in particular not to human conditions.

The mechanisms of oxPL toxicity in RAW264.7 cells were studied in more detail and seem to differ from the pathways observed in cultured smooth muscle cells. POVPC rapidly activated aSMase in cultured RAW264.7 macrophages. When aSMase activity was inhibited by NB19, cells became more resistant to POVPC-induced apoptosis, indicating that the activity of this enzyme is causally linked to POVPC toxicity in macrophages. Similarly, apoptosis induced by Jurkat cell-derived microparticles in RAW 264.7 macrophages was reduced when the cells were treated with an aSMase inhibitor [32]. However, it has to be emphasized that the (oxidized) lipid composition of microparticles is complex and therefore it is not possible to attribute toxic effects to discrete molecular species. PGPC showed a small inhibitory effect on aSMase after 15 minutes incubation. Data from preliminary experiments support the assumption that PGPC interferes with the enzymes of *de novo* ceramide synthesis localizing to intracellular membranes (ER), whereas aSMase likely generates ceramide in the plasma membrane (Koller D., Marlingapla Halasiddappa L., Hermetter A., unpublished). The obviously different roles of ceramide-generating pathways in PGPC- and POVPC-induced macrophage apoptosis are due to the small structural differences in the short-chain carboxylic acids that are the remnants of arachidonic acid oxidation in position *sn-*2 of the parent glycerophospholipids. PGPC contains a carboxylic function at the ω-position of the truncated acyl chain, whereas POVPC contains a chemically reactive aldehyde group. PGPC can only physically interact with the biomolecules in its immediate vicinity. It is rapidly internalized by the cells [33] and thus can influence the metabolic activities (ceramide synthesis) inside the cells. In contrast, POVPC is retained in the plasma membrane due to covalent Schiff base formation with the free amino groups of proteins and aminophospholipids [14,15]. This behaviour is in line with the observation that POVPC rapidly activates aSMase, which localizes at least in part to the cell surface. After long exposure time, POVPC can also reach the intracellular compartments where it modifies numerous cytosolic and membrane proteins [14]. In this regard, it behaves similarly as 4-hydroxynonenal (HNE) which is the fragment released from the ω-end of polyunsaturated fatty acids under oxidative stress [34]. Notably, HNE is more polar than POVPC and therefore partitions more easily into the cytosol and eventually the intracellular membranes where it covalently modifies proteins thereby triggering apoptosis, necrosis and inflammation [35,36] HNE is currently considered the major biomarker of lipid peroxidation [37].

In addition to diacylglycerophospholipids (mainly phosphatidylcholine), LDL also contains small amounts of alk(en)yl-acyl-analogs. Animal and human cell membranes, except the liver, may even contain large amounts of this phospholipid subclass [26,38-40]. Most alk(en)ylacyl phospholipid species (mainly containing phosphocholine- and ethanolamine head groups) also contain polyunsaturated fatty acyl chains in position *sn-*2 of glycerol and therefore are subject to modification by reactive oxygen species [11]. Here we show that the alkylacyl analogs of PGPC and POVPC are also potent inducers of apoptosis in RAW264.7 macrophages. The same effects were also observed in cultured vascular smooth muscle cells (unpublished). Currently, we do not know the reason for the higher ether lipid toxicities, which may be due to differences in biochemical stability and biophysical properties in the cell [41]. The *sn-*1 alkylether bond is resistant to hydrolytic cleavage and thus increases biochemical stability of the lipid molecule. From biophysical studies it is known that alkylacyl phospholipids are more densely packed [42] and increase the tendency of the membrane or some membrane areas to adopt nonbilayer lipid phases leading to membrane destabilization and alterations of membrane-associated enzyme functions.

One of the most striking phenomena of apoptosis is the controlled disintegration of cell (membrane) structures as a consequence of membrane blebbing, which is the release of microparticles (apoptotic blebs) into the immediate environments or the circulation. These particles are enriched in oxPL (mainly PGPC, POVPC and PEIPC) [20,43] and thus must be considered vehicles of toxic compounds that trigger detrimental effects far from the site of their formation. As a consequence they can propagate apoptotic and inflammatory processes within the same tissues or other organs [44,45]. The formation of microparticles may be favoured by the physical properties of the truncated phospholipids themselves [33] and/or the ceramide that is formed during the progress of apoptosis [46]. We found that PGPC leads to massive microparticle formation, whereas POVPC induced less vesicle release. The molecular shape of PGPC is conical and therefore it can directly induce high membrane curvature, a prerequisite for membrane vesiculation. The role of ceramide in PGPC-induced membrane blebbing is not obvious. PGPC seems to affect intracellular ceramide formation and to date it is unclear how the sphingolipid can reach the plasma membrane and contribute to cell surface-associated membrane effects under oxPL stress. A role of ceramide in POVPC-induced membrane blebbing is more plausible. The latter phospholipid stimulates aSMase and as a consequence ceramide can directly be generated in the plasma membrane. A direct contribution of POVPC to microparticle formation cannot be inferred from the currently available information. The preparation of typical lipid-lipid and lipid-protein conjugates of POVPC, which forms Schiff bases with free amino groups of proteins and aminophospholipids, is under way in our laboratory in order to characterize the biophysical properties and cellular effects of these membrane-associated components.

## Conclusions

In summary, PGPC, POVPC and their 1-O-alkyl analogs induce apoptosis in cultured macrophages with PGPC and ether-PGPC being more toxic than the POVPC counterparts. The higher toxicities are associated with more severe consequences to the cells. Loss of plasma membrane vesicles is more efficient if cells are exposed to PGPC. This result is physiologically highly relevant since apoptotic blebs contain large amounts of oxPL and carry these toxic compounds far away from the site of formation [20]. Thus the biological activity of oxPL is potentiated by this process. We conclude that the apoptotic effects of POVPC and its ether analog are coupled to the activity of aSMase. It remains to be elucidated, which pathway accounts for the formation of toxic ceramide under the influence of PGPC.

## Abbreviations

aSMase: Acid sphingomyelinase; BMM: Bone marrow-derived macrophages; DMEM: Dulbecco’s modified Eagle medium; E-PGPC: 1-O-hexadecyl-2-glutaroyl-*sn-*glycero-3-phosohcholine; E-POVPC: 1-O-hexadecyl-2-(5-oxovaleroyl)-*sn-*glycero-3-phosohcholine; EtOH: Ethanol; FACS: Fluorescence activated cell sorting; FCS: Fetal calf serum; JNK: c-Jun N-terminal kinase; LDL: Low-density lipoprotein; LPDS: Lipoprotein deficient serum; MAPK: Mitogen-activated protein kinase; MCSF: Macrophage colony-stimulating factor; mmLDL: Minimally modified low-density lipoprotein; MTT: 3-(4,5-dimethylthiazol-2-yl)-2,5-diphenyltetrazolium bromide; NBD: Nitrobenzoxadiazole; oxLDL: Oxidized low-density lipoprotein; oxPL: Oxidized phospholipids; PAF: Platelet-activating factor; PEIPC: 1- palmitoyl-2-(5,6-epoxyisoprostane)-*sn*-glycero-3-phosphocholine; PGPC: 1- palmitoyl-2-glutaroyl-*sn-*glycero-3-phosphocholine; PLPC: 1- palmitoyl-*sn*-glycero-3-phosphocholine; POPC: 1- palmitoyl-2-oleoyl-*sn*-glycero-3-phosphocholine; POVPC: 1- palmitoyl-2-(5-oxovaleroyl)-*sn-*glycero-3-phosphocholine; TLC: Thin-layer chromatography.

## Competing interests

The authors declare that they have no competing interests.

## Authors’ contributions

US was involved in study design, manuscript preparation and performed FACS analysis of cell death. ZAD performed viability and cell death measurements. DK measured enzyme activities. EZ synthesized phospholipids. H-P D synthesized enzyme inhibitors. EA and DK contributed cell biology. AH did the conceptual work and participated in study design and manuscript preparation. All authors read and approved the final manuscript.
